# A mixed methods partner-focused cost and budget impact analysis to deploy implementation strategies for school-based prevention

**DOI:** 10.1186/s43058-023-00511-6

**Published:** 2023-11-09

**Authors:** Andria B. Eisman, Jacob Whitman, Lawrence A. Palinkas, Judy Fridline, Christina Harvey, Amy M. Kilbourne, David W. Hutton

**Affiliations:** 1 Division of Kinesiology, Health, and Sport Studies, College of Education, Wayne State University, 2153 Faculty/Administration Building, 656 West Kirby Street, Detroit, MI 48202 USA; 2 Department of Economics, College of Liberal Arts, Wayne State University, 656 West Kirby Street, Detroit, MI 48202 USA; 3 School of Social Work, University of Southern California, 669 W 34th Street, Los Angeles, CA 90089 USA; 4 Genesee Intermediate School District, 2143 Maple Road, Flint, MI 48507 USA; 5 Oakland Intermediate School District, 2111 Pontiac Lake Road, Waterford Township, MI 48328 USA; 6 VA Ann Arbor Healthcare System, North Campus Research Complex, 2800 Plymouth Road, Bldg 16, Ann Arbor, MI 48109 USA; 7 Department of Health Management and Policy, School of Public Health, University of Michigan, M3525 SPH II, 1415 Washington Heights, Ann Arbor, MI 48109 USA

## Abstract

**Background:**

Obtaining information on implementation strategy costs and local budget impacts from multiple perspectives is essential to data-driven decision-making about resource allocation for successful evidence-based intervention delivery. This mixed methods study determines the costs and priorities of deploying Enhanced Replicating Effective Programs (REP) to implement the Michigan Model for Health™, a universal school-based prevention intervention, from key shareholder perspectives.

**Methods:**

Our study included teachers in 8 high schools across 3 Michigan counties as part of a pilot cluster randomized trial. We used activity-based costing, mapping key Enhanced REP activities across implementation phases. We included multiple perspectives, including state agencies, regional education service agencies, lead organization, and implementers. We also conducted a budget impact analysis (BIA, assessing the potential financial impact of adopting Enhanced REP) and a scenario analysis to estimate replication and account for cost variability. We used an experimental embedded mixed methods approach, conducting semi-structured interviews and collecting field notes during the trial to expand and explain the cost data and the implications of costs across relevant perspectives.

**Results:**

Based on trial results, we estimate costs for deploying Enhanced REP are $11,903/school, with an estimated range between $8263/school and $15,201/school. We estimate that adding four additional schools, consistent with the pilot, would cost $8659/school. Qualitative results indicated misalignment in school and teacher priorities in some cases. Implementation activities, including training and implementation facilitation with the health coordinator, were sometimes in addition to regular teaching responsibilities. The extent to which this occurred was partly due to leadership priorities (e.g., sticking to the district PD schedule) and organizational priorities (e.g., budget).

**Conclusions:**

Previous research findings indicate that, from a societal perspective, universal prevention is an excellent return on investment. However, notable misalignment in cost burden and priorities exists across shareholder groups. Our results indicate significant personal time costs by teachers when engaging in implementation strategy activities that impose an opportunity cost. Additional strategies are needed to improve the alignment of costs and benefits to enhance the success and sustainability of implementation. We focus on those perspectives informed by the analysis and discuss opportunities to expand a multi-level focus and create greater alignment across perspectives.

**Trial registration:**

ClinicalTrials.gov NCT04752189. Registered on 12 February 2021.

**Supplementary Information:**

The online version contains supplementary material available at 10.1186/s43058-023-00511-6.

Contributions to the literature

- Universal prevention in schools represents an excellent societal return on investment, but costs and resources remain a notable barrier to realizing its public health benefits.
- Research examining implementation costs and budget impacts from multiple perspectives aids in understanding the practical implications of deploying implementation strategies in community settings.
- Combining cost and qualitative data from multiple perspectives deepens our understanding of how resources and priorities are aligned—or not—that ultimately influence implementation success and sustainment.

## Introduction

Economic evaluation in implementation science has received increased attention as researchers, practitioners, and shareholders recognize that costs are foundational to successful implementation and sustainment. Practically speaking, costs and resources are essential reasons why implementation and sustainment efforts fail, including within educational settings [1, 2]. Although the focus on cost analysis and economic evaluation has expanded (e.g., [3–6]), cost data reports in implementation research remain limited [4, 7]. Cost is a crucial implementation outcome and may vary across multiple perspectives, including individuals, organizations, and systems [8]. Understanding the resources needed to achieve desired behavioral outcomes (e.g., reducing the initiation and escalation of substance use) is essential to successful implementation. However, most economic evaluation, including drug use prevention in schools, has focused on intervention costs and *not* the costs of implementation strategies required to deploy and sustain them [9]. Implementation costs and resources play a critical role in implementation success and may ultimately impact intervention costs (e.g., changes in student time engaging in the intervention) but are often ignored [3, 5, 7]. As a result, schools are often poorly equipped, from a resource perspective, to implement prevention effectively.

Systematic examination of costs for achieving program outcomes using implementation strategies is vital for evidence-based intervention (EBI) sustainability in schools as in other settings [9]. Organizations benefit from evidence that supports (or refutes) investment in specific strategies as an efficient use of resources. Pragmatic cost data can help prioritize implementation efforts, including proactively allocating resources for this purpose and informing implementation strategy deployment in resource-sensitive ways [8–10]. Enhanced Replicating Effective Programs (REP; see Table 1 for standard and Enhanced REP components), for example, has demonstrated effectiveness in improving intervention uptake and has been deployed in community and school settings [11–13] but also has cost implications [14]. The standard implementation of MMH is consistent with REP as it includes the curriculum, standard training, and as-needed technical assistance. Enhanced REP is a more intensive strategy and comprises three primary components: (1) curriculum/packaging tailoring, (2) tailored training to meet context-specific needs (e.g., trauma-skilled approaches), and (3) implementation facilitation (IF), hands-on guidance, and support for implementation by trained facilitators [15]. Implementation strategies, such as Enhanced REP, require resources above and beyond standard implementation. Estimating costs is a vital first step in ensuring that the school community and shareholders obtain the highest value from implementation efforts.

**Table 1:** Standard implementation and enhanced replicating effective programs (Enhanced REP) components for Michigan Model for Health™ (MMH) implementation (adapted from Kilbourne et al. 2015)

Component		Standard Implementation	Enhanced REP
Package	MMH curriculum manual		Intervention manuals customized to low-resource settings based on population needs and setting resources using input from an advisory board
Training	Standard MMH training		Customized training based on input from package step above
Implementation facilitation (IF)	As needed technical assistance with program delivery		IF components: Review potential barriers and set goals based on barrier assessment, provide specific implementation guidance and facilitate information sharing and long-term plans for sustainability, meet regularly with providers, align intervention with organization, and advocate for implementation to leadership

**Fig. 1:**
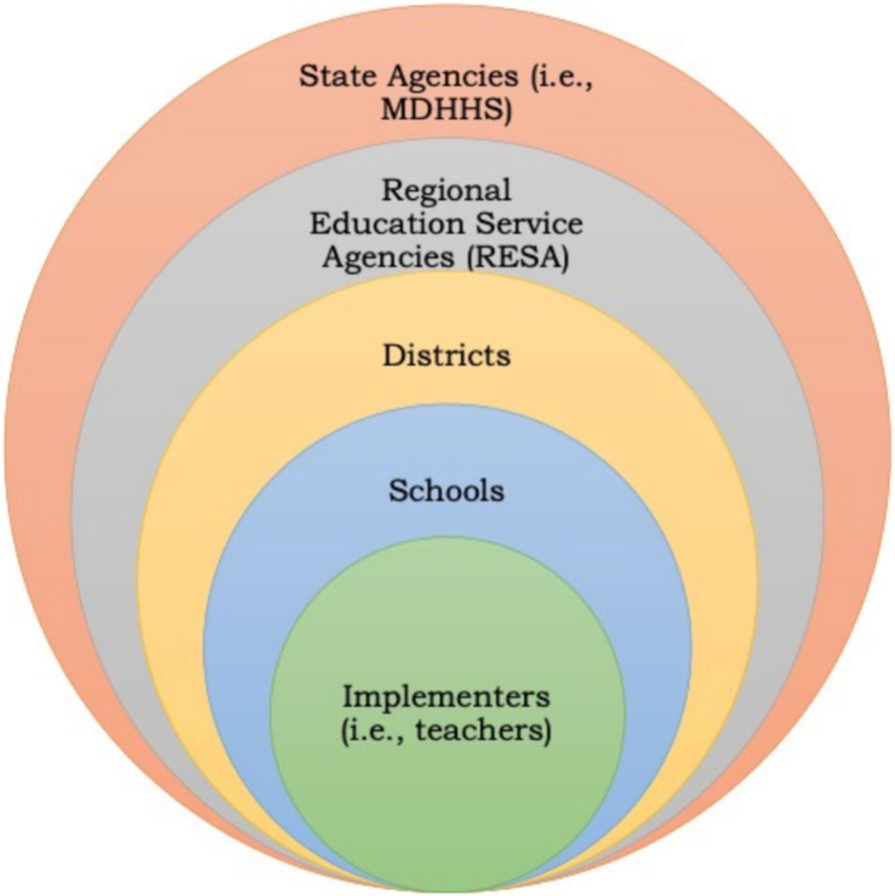
Multi-level perspectives on implementation costing in educational settings (informed by [18, 19, 21])

Limited research has focused on practical information on implementation costs across essential partner perspectives, including within the education sector (see Fig. 1). While an aggregated (e.g., societal) perspective remains the gold standard in economic evaluation, it may not provide sufficient information for understanding why implementation fails to achieve desired objectives [16, 17]. Determining implementation strategy costs from multiple perspectives is essential to data-driven decision-making about resource allocation for EBI implementation. The study perspective is the *key determinant* of which costs researchers will include in an economic evaluation [17]. The perspectives included reflect value judgments within an economic evaluation as those included are considered the most relevant [17]. Figure 1 illustrates various levels of shareholders (i.e., the interested parties) that influence implementation in schools, adapted from Ferlie and Shortell [18] and informed by other work in the education sector [19, 20]. The levels include state agencies, regional education service agencies (RESAs), school districts, school buildings, and classrooms/teachers. Each level may play a role in the intervention and implementation resource availability and use. For example, the state agency may support initial intervention development, updates, and implementation startup (i.e., training). The RESAs may determine to what extent implementation supports (via their health coordinator staff) are provided, from as-needed technical assistance to hands-on implementation facilitation (IF), depending on their budget and other initiatives. School districts will choose whether or not to adopt the intervention and permit teachers the time to attend training and utilize the technical assistance, depending on the school’s needs, staffing, and competing demands. Finally, the teacher or implementer, depending on priorities and resources at other levels, may need to decide whether or not to adopt the intervention and engage in training and support and, if so, at what cost (i.e., opportunity cost- time they could have spent doing another productive activity or engaging in leisure time). Additional research is needed to understand differing resource burdens and benefits across levels, as this is essential to effectively implementing and sustaining interventions in community settings [16].

A mixed methods approach to economic evaluation enables a comprehensive assessment of cost and resource burdens across shareholder levels as, undoubtedly, the levels are interdependent [16, 22]. We include qualitative data to contextualize the quantitative cost data and better understand multi-level perspectives, including priorities, resource burdens, and potential alignment and misalignment across these levels [22]. Qualitative data are fundamental in understanding implementation costs because the *actual* costs depend on the context in which intervention implementation happens [22]. Our objectives in this pilot cluster RCT (randomized controlled trial) mixed methods cost analysis were as follows: (1) estimate the costs of deploying a multi-component implementation strategy (Enhanced Replicating Effective Programs) from critical shareholder perspectives to implement an evidence-based universal prevention intervention, the Michigan Model for Health™ (MMH); (2) understand context-related costs and priorities that influence Enhanced REP deployment for MMH implementation; (3) estimate the potential budget impact of deploying Enhanced REP for MMH and similar health curricula in other school environments, that is, estimating replication costs informed by qualitative data.

## Methods

### Overview

**Fig. 2:**
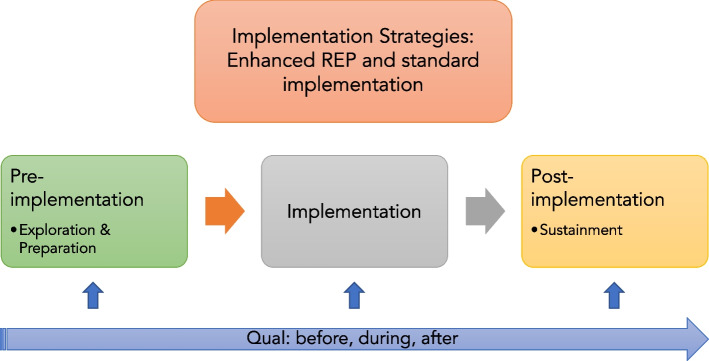
Experimental embedded mixed methods design

The current study focuses on reporting costs related to deploying implementation strategies for the Michigan Model for Health™ universal prevention curriculum as part of a group RCT. Specifically, we focus on quantifying the incremental implementation costs of deploying the Enhanced REP implementation strategy bundle. We use an experimental embedded mixed method design to obtain a more in-depth understanding of the results (see Fig. 2), focusing on applying qualitative results to support the expansion and explanation of quantitative results. We also estimate the costs of replicating Enhanced REP for universal prevention in other school settings by determining the budget impact of reallocating costs to likely interested parties based on shareholder input.

### Setting and participants

We included data from a randomized controlled trial in 8 schools across 3 Michigan counties during the 2021–2022 school year: 4 randomized to Enhanced REP (see Table 1 for Enhanced REP components) and 4 to standard implementation. Schools varied in size, student demographics, geographic area (e.g., urban, rural, suburban), and economic characteristics. These characteristics guided randomization to ensure balance across study conditions, with all participating schools having at least 20% of students qualify for free-and-reduced lunch. These criteria supported a diverse student population, including those at higher risk of poor health outcomes, as economic disadvantage is a robust risk factor [23]. Two school health coordinators serve the study counties and provide implementation support for MMH consistent with REP. School health coordinators in Michigan may serve one or more counties depending on the population and geographic dispersion. Each health coordinator in the study served four participating schools. We focus on the incremental costs to deploy Enhanced REP. This study centered on high school, as youth are at increased risk for substance use onset and escalation [24].

### Program Components

#### Implementation strategy bundle: Enhanced REP

Table 1 provides an overview of standard and Enhanced REP. REP is a multi-component strategy focused on enhancing the fit between the intervention and context while maintaining fidelity to core EBI functions. It is based on the CDC’s research-to-practice framework [13, 25] and guided by Social Cognitive [26] and Diffusion of Innovations Theories [27]. The standard implementation of MMH includes three components: curriculum packaging materials, teacher training, and as-needed technical assistance from regional school health coordinators. Standard implementation is consistent with standard Replicating Effective Programs: REP. However, low-level implementation strategies such as REP are not always sufficient to implement complex behavioral interventions effectively [12]. Researchers developed a more intensive version of REP (Enhanced REP) that includes tailoring curriculum materials and training and ongoing provider consultation or implementation facilitation (IF). IF is based on iPARIHS: integrated Promoting Action on Research Implementation in Health Services framework [12, 28]. Researchers have found improved program fidelity in clinical research among sites deploying Enhanced REP [29]. More intensive strategies, however, are also generally more expensive. Implementation costs fundamentally influence program delivery in schools with multiple competing demands and scarce resources [9, 30]. Systematic examination of costs for multi-component implementation strategies is vital to provide pragmatic information related to resource decision-making, scale-up, and sustainability in community settings [9, 14].

#### Intervention: Michigan Model for Health™ (MMH) curriculum

MMH is a universal prevention curriculum that has demonstrated efficacy in reducing substance use and improving adolescent mental health outcomes [31, 32]. The curriculum is theoretically based, grounded in Social Cognitive Theory [33] and the Health Belief Model [34]. The MMH curriculum is recognized as evidence-based by CASEL (the Collaborative on Academic and Social and Emotional Learning) and aligned with Michigan and National (United States) Health Education standards [35, 36]. The curriculum is widely adopted across Michigan; 91% of health teachers use MMH [37] MMH, however, is infrequently delivered with fidelity [37, 38]. A statewide study found that 58% of educators failed to meet state-identified fidelity standards [37] (i.e., delivering 80% or more of the curriculum); among schools in economically challenged communities, the proportion increased to 73% [38]. The trial focused on piloting Enhanced REP to improve MMH implementation. Fidelity challenges are not unique to MMH, as most other school-based universal prevention interventions are delivered suboptimally [39].

In Michigan, MMH is widely adopted in schools throughout the state. Multiple shareholders are involved in its implementation, including the state agencies, the Michigan Department of Health and Human Services (MDHHS) and Department of Education (MDE), the RESAs (Regional Education Service Agencies) through the health coordinators and the teachers who receive the curriculum, training, and support for MMH from the health coordinator in their region. Other levels, including districts and local school buildings, vary in intensity, investment, and active versus passive involvement in the health curriculum (see Fig. 1). Determining disaggregated costs across all levels is time- and resource-intensive and thus not always feasible. In the current study, we adopt a pragmatic approach to costing MMH implementation using Enhanced REP and focus on those levels we expect to use these results to inform decision-making and resource allocation. We also incorporate mixed methods to account for considerations across all levels.

### Procedures

The study initially included ten schools randomized into control and intervention sites (0,1). We had schools currently using MMH that failed to meet state standards for implementation (delivering less than 80% of the curriculum) and/or reported one or more barriers to MMH implementation from a list of 10 common barriers to participation in the study. We checked randomization at the control/intervention group level and then by the specific school representations across the number of students (i.e., school size, free and reduced lunch status, and proportion of minority students).

### Estimating costs

We used an activity-based micro-costing approach mapping key activities of Enhanced REP across implementation phases. We adopted multi-level perspectives, including state agencies (e.g., MDHHS), regional education service agencies (RESA), lead organization (e.g., university), and implementers (e.g., teachers). We estimated the incremental implementation strategy costs during the trial. We also conducted a budget impact analysis using explicit, real-world costs to assess the economic impact of replication and considering the cost distribution across levels in the educational system. We focused on these perspectives as they are consistently those perspectives that are explicitly involved in decision-making and resource allocation for health education. We used the EPIS (Exploration, Preparation, Implementation, and Sustainment) framework to guide implementation phases [40]. This approach is consistent with other costing methods for implementation strategies, including the COINS (Costs of Implementing New Strategies) model [41], and EPIS has been used previously to guide determining implementation strategy costs [4, 5]. See Table 2 for implementation strategy activities across EPIS phases. We assigned a cost to each activity throughout the implementation process, also called a shadow price, as it does not incur an actual outlay of additional compensation [42]. To accurately assess the time spent on each activity, and therefore the ingredient’s cost, individuals (health coordinators, research staff) recorded time spent on tasks throughout implementation strategy deployment and documented those activities using an online activity log, including identifying the individuals involved. For example, we tracked the time health teachers spent in IF activities using the health coordinator’s log that included this information.

**Table 2:** Pilot RCT cost inputs

**Phases**	**Activities**	**State**		**RESA**		**University**		**Implementer**		
		Hours	Cost	Hours	Cost	Hours	Cost	Hours	Cost	Total Costs
**Exploration**	*Engagement & Assessment *									
	Meetings with health coordinators	-	-	12	$446	6	$164	-	-	$610
	Assessing student needs	-	-	-	-	20	$1,718	-	-	$1,718
	Assessing existing efforts	-	-	-	-	15	$409	-	-	$409
	*Feasibility*									
	Assessing preliminary feasibility	-	-	-	-	10	$237	-	-	$237
	Assessing project alignment with current efforts	32	$1,863	-	-	16	$1,374	-	-	$3,237
	Health coordinator meetings with ISD leadership	-	-	4	$228	16	$436	-	-	$664
	**Total Exploration **	32	$1,863	16	$674	83	$4,338	0	-	**$6,875 **
**Preparation**							Exploration cost per school			$1,719
	*Planning & Preparation*									
	Logistics planning	-	-	32	$1,190	32	$873	-	-	$2,062
	Health coordinator facilitation training preparation	-	-	-	-	13	$864	-	-	$864
	Health coordinator training: Facilitation	-	-	-	-	21	$1,276	3	$230	$1,506
	*Recruitment*									
	Identify and recruit eligible schools	3	$112	52.75	$1,961	27	$1,147	8	$571	$3,790
	Prepare for package dissemination	-	-	-	-	15.5	$423	-	-	$423
	Graphic Design Assistance & Website Building	-	$8,090	-	-	30	$712	-	-	$8,802
	**Total Preparation **	3	$8,202	84.75	$3,150	138.5	$5,294	11	$801	**$17,448 **
**Implementation**							Preparation cost per school			$4,362
	*Teacher Training*									
	Teacher Training	-	-	36.25	$1,347	-	-	78	$5,981	$7,329
	Lesson Preparation & Action Planning	-	-	-	-	-	-	30.5	$2,339	$2,339
	*Management*									
	Implementation facilitation (teacher meetings)	-	-	25	$929	-	-	25	$1,917	$2,846
	Facilitation support for health coordinators	-	-	23	$855	23	$1,301	-	-	$2,156
	Website Refinement	-	$4,964	-	-	-	-	-	-	$4,964
	**Total Implementation **	0	$4,964	84.25	$3,132	23	$1,301	133.5	$10,237	**$19,634 **
**Sustainment**							Implementation cost per school			$4,909
	*Refinement *									
	Assessment/Evaluation	-	-	10	$372	-	-	-	-	$371.72
	Annual training updates	-	-	19.25	$716	-	-	-	-	$715.56
	Facilitation (light touch version)	-	-	14.75	$549	-	-	9	$690	$1,238.44
	Packaging refinement	-	$1,331	-	-	-	-	-	-	$1,331.00
	**Total Sustainment**	0	$1,331	44	$1,636	0	-	9	$690	**$3,657 **
							Sustainment cost per school			$914
**Totals**		**35**	**$16,360 **	**229**	**$8,591 **	**244.5**	**$10,934 **	**153.5**	**$11,729 **	**$47,614 **
							**Total Cost per school**			**$11,903 **

**Note:** We rounded to the nearest dollar for the sake of clarity in reporting. As a result, some totals in the table may be subject to minor discrepancies due to rounding. These discrepancies do not significantly impact the overall findings and conclusions of this study. **Sources:** Bureau of Labor Statistics, Mackinaw Policy Center. Time estimations are based on reported activity logs. Wage data includes Full-Time Equivalency for estimated benefits for all personnel. Wage data for the State Agency Employees & Health Coordinators is derived from the Mackinaw Policy Center. All remaining wages are derived from the Bureau of Labor Statistics (BLS) 2021 Occupational Employment and Wage Statistics report for the state of Michigan.

We assigned monetary values to each activity or other input upon establishing necessary implementation resources (i.e., activities) across phases. Most resource costs related to the Enhanced REP implementation strategy were personnel, specifically implementers, implementation support professionals, and coordinating organization time. We include costs within the education sector, including fixed (e.g., startup costs such as exploration and preparation activities involved in replication) and variable costs (e.g., personnel time spent on the provision of implementation facilitation), and time outside the education sector (e.g., teacher personal time costs or personal opportunity costs) when these could be reasonably estimated for a pragmatic evaluation of costs in this analysis. Consistent as described by Gold and colleagues, opportunity costs refer specifically to the value of resource inputs in their next best alternative use [3]. Opportunity costs do not represent a financial cost per se but rather ascribe a value to the “something given up.” In the case of this pilot study, the “something given up” is generally time. Often, the time is personal or leisure outside of typical work hours. For this reason, we use the term personal opportunity cost to represent the valuation of the time costs outside the education sector spent by providers to engage in implementation strategy activities [42].

To properly micro-cost, we assessed the amount of time spent on each activity and the costs associated with each person’s time. To optimize applicability across contexts, we utilized salary range information from the Bureau of Labor Statistics (BLS) 2021 Occupational Employment and Wage Statistics report for the state of Michigan [43]. Since the BLS does not report wage data for the health coordinators (i.e., implementation support professionals) or state agency partners, we take the average salaries from the Mackinac Policy Center’s Michigan Government Salary [44] database. We used median (50th percentile) salaries from administrative data as our base case. We used median salaries to reduce the bias that the school districts involved in the project are subject to abnormal labor market conditions.

The time horizon for the current pilot study is aligned with the experimental design, one academic year. For the Sustainment phase, we projected the recurring costs of the program over time for each additional semester, following the first semester among teachers who implemented for two semesters.

### Cost analysis plan

We estimated the labor cost, including fringe benefits by multiplying each hourly rate by 1.34, to reflect the actual time costs [43]. We used the BLS’s Employer Cost for Employee Compensation from June 2021 to inform the fringe rate. According to BLS Health Education Professionals (Primary, Secondary, and Support), the non-salary benefits are estimated to be 34% of the annual salary [45]. Following the BLS’s method, we divided annual salaries for each occupation by 2080 to calculate the hourly rates for each occupation and included fringe benefit costs. However, for teachers, we follow Michigan’s Public School Reporting Units’ Reporting Instruction Manual for K-12 and ISD teachers and divide annual salaries by 1280 h per year [46]. This analysis uses costs from 2021, the most up-to-date data available.

Our analysis also stratified relative costs by perspective groups— the agents involved in funding and deploying the implementation efforts [47]. The perspectives included are the implementer, the RESA, and the state agency (e.g., MDHHS). We estimate costs for each perspective group to identify respective cost burdens in implementing interventions. Ideally, an implemented program makes financial sense for each group to support fidelity and sustainment. In this way, each perspective group will understand the amount of investment they have in the program’s success and can identify priorities and forecast budget and labor requirements accordingly.

We conducted a scenario analysis to characterize uncertainty in the cost estimates in a broad sense by estimating the “best” and “worst” case scenarios [48]. This approach is suitable given data limitations related to a pilot trial [49]. Since cost analysis cannot produce significance testing, a scenario analysis accounts for future costs’ potentially high variability (i.e., uncertainty) to inform decision-makers. We conducted the scenario analysis using the 90th and 10th percentile wages according to the State of Michigan’s Bureau of Labor Statistics 2021 Occupational Employment and Wage Statistics report. The Mackinaw Policy Center reports Michigan public employee compensation and, therefore, does not include ranges of salaries. To circumvent this dilemma, we averaged the percentage increases over the median from the BLS’s salary reports, 44.7%, and applied that increase to the salaries found in Mackinaw Policy Center’s database. We used the same approach for the 10th percentile, applying a decrease of 39.7%.

### Budget impact analysis plan

We conducted a budget impact analysis to estimate the potential budget impact for replication. While traditional BIA may imply adopting an organization perspective, the practical application of BIA in implementation science has expanded this to include other perspectives that would benefit from receiving information about the potential budget impact from these various perspectives. A recently published budget impact tool, for example, permits users to customize the BIA by selecting the resources that each user group (i.e., perspective) would require for a delivery model for MOUD (medication for opioid use disorder) [50]. Wagner and colleagues also discuss different perspectives for BIA in implementation science [51]. While costs such as website design and the creation of materials were fixed, the bulk of costs involved in deploying the implementation strategy (Enhanced REP) were variable. Since the initial coordinating body was researchers at a university who would not participate in replication, we also evaluated which tasks originally completed by researchers would be relinquished to other parties. We identify the appropriate responsible party in coordination with the state agency and RESA partners.

### Qualitative data collection

Our interview guide was informed by the Enhanced REP components that align with EPIS constructs in the outer context (e.g., intervention recipient needs/characteristics), innovation factors (the MMH intervention and fit with the context), the inner context (i.e., school setting), and multi-level constructs such as barriers and facilitators and resources needed and utilized [40]. We (PI or project coordinator) conducted semi-structured interviews with study teachers, with suggested follow-up probes based on question responses. We conducted pre-, interim-, and post-implementation interviews, which were recorded and transcribed verbatim. We conducted pre-, interim-, and post-implementation interviews, which were recorded and transcribed verbatim. We also included field notes taken by the research team during implementation support practitioner (i.e., health coordinator) meetings with the research staff and health coordinator notes from meetings with teachers to supplement the interviews.

### Qualitative data analysis

We used rapid qualitative data analysis techniques [52, 53], including structured template data summaries and data matrix displays to compile the data from the implementers over time. The rapid analysis approach aims to generate reliable research findings that can be applied to practice as soon as possible. In addition, researchers have found that rapid analysis findings generate reliable, timely information similar to other qualitative approaches such as thematic analysis [54]. We followed the procedure for rapid analysis as described by Koenig et al. [55]. We summarized interview responses using a multistep method. First, four team members read through each transcript and related field notes, noting vibrant responses, defined as contextually meaningful and detailed responses. Second, the research team developed and tested a structured template to standardize how discussion content was captured. Specifically, based on the interview guide, we identified key topics or themes from the interviews related to implementation costs as broad categories for the summaries. Three team members tested the template with a single discussion, compared summaries, and resolved discrepancies to standardize the summary procedure. Third, after summarizing the interviews using the template, we aggregated the data across participant groups and time points to create a comprehensive longitudinal matrix. Longitudinal data matrices help researchers understand how participants’ experiences may change over time [56]. This longitudinal data matrix summary facilitated a systematic comparison between experimental groups [57]. We identified the key themes relevant to costs and resource needs to aid in contextualizing the results for each teacher over the semester. Next, we reviewed field notes from the IF meetings during the preparation, implementation, and sustainment phases and added relevant cost concepts to the costs and resource needs to the summary.

### Mixed methods: data integration

The data integration centers on using qualitative data for explanation and expansion. We applied qualitative results to help explain the quantitative results (e.g., costs), including placing the costs across shareholder groups in context and providing a broader explanation of the costs and benefits of deploying Enhanced REP over a school year. For expansion, we used qualitative results to deepen our understanding of what costs are not sufficiently captured with micro-costing, such as personal time costs (e.g., personal time outside the education sector that reflect opportunity costs for participating in Enhanced REP) and areas of alignment and misalignment across groups in costs and benefits of implementation. Thus, we focus on comprehensively understanding cost and resource considerations and expand on the quantitative findings [58].

## Results

Two schools dropped out early in the study, leaving eight for our cost analysis and 4 for the Enhanced REP cost estimates. Of the participating schools, 6 had student populations over 1000, equally divided between control and trial groups. The smallest participating school had 213 students and the largest had 2051 students.

Based on publicly available school data [59] (Additional file 1), participating schools averaged a free-and-reduced lunch rate of 43.4%, ranging between 23.4 and 88.6%. Three schools crossed the 70% threshold to indicate high levels of free-and-reduced lunch participation, two in the treatment group and one in the control group. Six participating schools had white students as the majority racial demographic in their schools. On average, 62% of the participating schools’ students were white, followed by black (27%), Latinx (4%), mixed (4%), Asian (3%), American Indian (0.1%), and Native Hawaiian (0.05%). We found no indication to suggest heterogeneity between the trial and control groups based on racial demographics or socioeconomic status based on these data [59]. We include information on school demographics and tests for heterogeneity in the Additional file 1.

### Cost data

Table 2 includes the estimated costs for each activity across implementation phases and the total cost per site (i.e., school). During the exploration phase, costs totaled $1781 per school. The preparation phase cost $4362 per school, and the implementation phase costs were $4909. The sustainment phase of the Enhanced REP implementation will have an estimated annual cost of $914 per school. The total cost for the four-school implementation of Enhanced REP was $47,614. MDHHS and RESAs bore $16,360 or 34.4% of the total cost, followed by the implementers with $11,729 (24.6%), the university (lead agency) with $10,934 (23.0%), and the RESA with $8591 (18.0%) respectively.

Table 3 includes the scenario analyses. Utilizing the higher ranges (the “worst case” range), we estimated a total cost of $60,804 for the four schools or $15,201 per school. This was a total increase of 27.7% from our baseline cost model. Utilizing the lower range (the “best case” range), we estimated a total cost of $33,056 for the four schools or $8264 per school. This was a total decrease of 30.6% from our baseline cost model, generating a range of potential costs.

**Table 3:** Scenario analyses

**Low/high salary estimates**	**Shareholder group**				
10th percentile salaries	State	University	RESA	Teacher/ implementer	Totals
Exploration	$1,123	$2,023	$366	-	$3,512
Preparation	$8,149	$2,984	$1,656	$496	$13,285
Implementation	$4,964	$583	$1,646	$6,441	$13,634
Sustainment	$1,331	-	$860	$434	$2,625
Total	$15,567	$5,590	$4,527	$7,372	**$33,056 **
Cost/school					**$8,264**
90th percentile salaries					
Exploration	$2,621	$6,423	$892	-	$9,936
Preparation	$8,235	$7,675	$4,099	$1,135	$21,144
Implementation	$4,964	$20,271	$2,539	$15,383	$43,156
Sustainment	$1,331	-	$2,128	$966	$4,425
Total	$17,151	$16,030	$11,193	$16,429	**$60,804 **
Cost/school					**$15,201**

**Note:** We rounded costs in the table to the nearest whole number for the sake of clarity in reporting. As a result, some totals in the table may be subject to minor discrepancies due to rounding. These discrepancies do not significantly impact the overall findings and conclusions of this study

### Budget impact analysis

Table 4 includes results from a BIA to estimate the budget impacts of deploying Enhanced REP in new school environments (e.g., replication). The initial coordinators for Enhanced REP deployment were a group of researchers at a university. These researchers would participate as consultants for future replication but will not be coordinating future implementations. To develop an estimate for the replication costs, we assessed which tasks originally completed by researchers would be relinquished to either the school district, state health department, or regional health coordinators within RESAs. This was an essential aspect of our incremental cost analysis. We identified the appropriate responsible party in coordination with agency partners’ expert opinions. For example, during exploration, our partners indicated that assessing student needs would likely be relinquished to the RESA. During preparation, the health coordinator IF training would likely be primary coordinated by the state agency, with additional RESA involvement. Our replication cost methods also remove the fixed costs associated with implementation, such as designing materials and building the website. With fixed costs deducted, all remaining costs are the employee time costs.

**Table 4:** Budget impact analysis results

**Phases**	**Description**	**State**		**RESA**		**University**		**Implementer**		
		Hours	Cost	Hours	Cost	Hours	Cost	Hours	Cost	**Total Costs **
**Exploration**										
	*Engagement & Assessment *									
	Meetings with health coordinators	6	$349	6	$223	-	-	-	-	$572
	Assessing student needs	-	-	20	$1,532	-	-	-	-	$1,532
	Assessing existing efforts	-	-	15	$1,149	-	-	-	-	$1,149
	*Feasibility*									
	Preliminary feasibility considerations	-	-	10	$766	-	-	-	-	$766
	Assess alignment with current efforts	32	$1,863	16	$932	-	-	-	-	$2,795
	Health coordinator meetings with ISD leadership	-	-	20	$1,453	-	-	-	-	$1,453
	**Total Exploration **	38	$2,213	87	$6,054	0	-	-	-	**$8,266 **
							Exploration cost per school			$2,067
**Preparation**	*Planning & Preparation*									
	Logistics planning	-	-	48	$3,045	-	-	-	-	$3,045
	Health coordinator facilitation training prep	4	$233	8	$455	-	-	-	-	$688
	Health coordinator training: Facilitation	9	$524	8	$297	4	$344	3	$230	$1,395
	*Recruitment*									
	Identify and recruit eligible schools	3	$112	67.75	$2,715	-	-	8	$571	$3,398
	Prepare for package dissemination	-	-	15.5	$576	-	-	-	-	$576
	**Total Preparation **	16	$868	147.25	$7,089	4	$344	8	$801	**$9,103 **
							Preparation cost per school			$2,276
**Implementation**	*Teacher Training*									
	Teacher Training	-	-	36.25	$1,347	-	-	78	$5,981	$7,329
	Lesson Preparation & Action Planning	-	-	-	-	-	-	31	$2,339	$2,339
	*Management *									
	Implementation facilitation (teacher meetings)	-	-	25	$929	-	-	25	$1,917	$2,846
	Facilitation support for health coordinators	11.5	$670	11.5	$427	-	-	-	-	$1,097
	**Total Implementation **	11.5	$670	72.75	$2,704	0	-	133.5	$10,237	**$13,611 **
							Implementation cost per school			$3,403
**Sustainment**	*Refinement *									
	Assessment/Evaluation	-	-	10	$372	-	-	-	-	$372
	Annual training updates	-	-	19.25	$716	-	-	-	-	$716
	Facilitation (light touch version)	-	-	14.75	$548	-	-	9	$690	$1,238
	Packaging refinement	-	$1,331	-	-	-	-	-	-	$1,331
	**Total Sustainment **	0	$1,331	44	$1,636	0	-	9	$690	**$3,657 **
							Sustainment cost per school			$914
** Totals**		**65.5**	**$5,082 **	**351**	**$17,483 **	**4**	**$344 **	**151 **	**$11,729 **	**$34,637 **
							**Cost per additional school **			**$8,659**

**Note:** We rounded costs in the table to the nearest whole number for the sake of clarity in reporting. As a result, some totals in the table may be subject to minor discrepancies due to rounding. These discrepancies do not significantly impact the overall findings and conclusions of this study. **Sources:** Bureau of Labor Statistics, Mackinaw Policy Center. Time estimations are based on reported activity logs. Wage data includes Full-Time Equivalency for estimated benefits for all personnel. Wage data for the State Agency Employees & Health Coordinators is derived from the Mackinaw Policy Center. All remaining wages are derived from the Bureau of Labor Statistics (BLS) 2021 Occupational Employment and Wage Statistics report for the state of Michigan

From the educational system perspective, we estimate the budget impact for Enhanced REP of $34,637 to add four additional schools or $8659.17 per school. We estimate only the budget impact using the same number of schools in the pilot test. Considering other schools would require speculation on the economies (or diseconomies) of scale of the implementation. We also calculated budget impacts from each shareholder’s perspective when estimating the BIA. The university tasks were transferred to the health coordinators and state agency employees, with schools bearing no additional cost in replication. This determination was based on input from the state agency and RESA partners on what types of costs they typically incur related to implementation, although it may vary. Consistent with the current partnership structure, University employees would serve only in minor consultation roles with an estimated cost of $343.53. We anticipated that school districts would have the most significant increase in costs and time commitment. We estimated a total cost of $17,483 for the school districts, an increase of $8892 or roughly doubling the cost. State agency partners funded all the fixed costs in implementation. Thus, we estimated a decrease in their share of the overall cost in the budget impact analysis. Simultaneously, the state agency would likely contribute more labor efforts to repeated implementation. For the state agency partners, we estimated a decrease in fixed costs of $14,385 and an increase in variable labor costs of $3107, resulting in a net cost reduction of $11,278.

Since the estimated budget impacts of replication in new schools are all employee time costs, we may also consider these to be changes in total labor hours. We anticipate an increase in labor provided by the state from 30.5 to 65.5 h and an increase in the district’s efforts from 229 to 351 h to cover the decrease in the university efforts. In total, we estimate a net reduction of 91 h in labor related to the budget impact of replication.

### Qualitative data

Teacher interviews aided in putting costs in context with quantitative data. We report three interrelated central themes. The themes include (1) priority and resource alignment across perspectives, (2) the impact of implementation leadership, and (3) the influence of collaboration and support on the implementer (i.e., teacher) burden.

#### Priority and resource alignment across perspectives

This theme refers to the extent to which priorities and resources are aligned or shared across implementer, organizational leadership (i.e., school), and district or RESA (e.g., implementation climate; [60]). Qualitative data indicated that, in some schools, there was a misalignment between teacher and district professional development (PD) priorities and policies, posing challenges to curriculum-specific PD and activities. One health teacher reported, for example, that the district predetermined their PD time and did not necessarily align with health or health priorities. “(Our PD) gets lumped in with (other topics), and we don’t get to separate.” She stated that she completed any curriculum-focused PD during non-school hours. Another teacher reported notable challenges engaging in MMH curriculum or health-specific trainings due to not obtaining coverage for her classes. “We couldn’t even get subs when we were sick, so they definitely didn’t give me time to work on this curriculum, so I couldn’t take a half day to work on it or anything like that… but I think that’s also because the school … our administrators were so overloaded with work too.”

Related, teachers shared instances when they saw misalignment between school, teacher, RESA/implementation support professionals (i.e., health coordinators) priorities, and resource allocation. One teacher expressed frustration with allocating significant time and resources to the “next new thing” versus investing in evidence-based health curricula that have already been adopted. For instance, why aren’t schools asking, “what can we do to help the *existing* (foundational Tier 1) programs that are already there (like MMH) because we (can) create stronger and deeper ties (with students) that are going to last more than the time period of the grant or that little bit of money this (new) initiative goes for.” Another teacher stated that this cycle of continuously adopting new programs was deeply engrained, and some school environments are very “resistant to change.” Teachers reported that if they did want more curriculum-specific focus, they had to find time outside of school hours. Thus, some teachers’ time to engage with health coordinators and receive IF related to the MMH updates was primarily during personal time. Other teachers attempted to consult with health coordinators during their planning hour, but this was frequently disrupted due to other school priorities (e.g., covering other classes). As a result, some teachers’ capacity to engage with IF was inconsistent, and health coordinators spent notable time trying to schedule and reschedule meetings with implementers (i.e., teachers); the misalignment created additional barriers and resulted in significant opportunity costs within and outside the education sector, for teachers and health coordinators.

In cases of alignment between the district, teacher (implementer), and school priorities and resources, the implementer reported *reduced* time in curriculum prep versus standard MMH implementation. She reported she was able to spend more time on activities to enhance student engagement with the curriculum, build connections, and meet the diverse needs of students. A teacher in this scenario reported having regular meetings with the health coordinator in which she was able to obtain both relational and informational support. “(The health coordinator) is quite helpful. She’s always given me tips additional resources that might be helpful, not just for me, but for students and their families, so we’ve been meeting every (week) to check in and share how things are going, and so it’s been very valuable having her as a resource.”

#### The impact of implementation leadership

Implementation leadership refers to specific leadership behaviors that are likely to advance EBI implementation efforts; it can include different dimensions, such as proactive (e.g., getting ahead in problem-solving implementation challenges) and supportive (e.g., encouraging staff and providing assistance) leadership [61]. Teachers in the current pilot reported stark contrasts in how much their leadership aided in setting the groundwork for successful implementation. In one case, the teacher said that “school leadership gave me the autonomy to do whatever I needed to do (to participate in the project),” an example of supportive leadership behavior. Another teacher reported that her school leadership gave her the “green light” but also made it clear that there would be no provisions for her participation. Nothing explicitly supported access to resources and responsibility adjustments to enhance the likelihood of successful MMH implementation. “What did I actually get support-wise from (admin)? Nothing.” In observing the profound differences between the settings, the health coordinators remarked that working with administration ahead of time to make EBI implementation more of a priority is an essential next step to improve the alignment of priorities and resources to support teachers.

#### The influence of collaboration and support on implementer burden

This theme centers on the extent to which the school environment permits engagement with peers and implementation support personnel to reduce individual implementer burden and enhance the overall implementation of the curriculum. For teachers who work with other health teachers in their school building, collaborating—or not—may profoundly impact the implementer’s workload and capacity to engage in implementation strategies. One teacher reported that her district requires a common final exam despite many of her peers *not* utilizing evidence-based health curricula. “(I) still had to teach parts of what (others in her building were) teaching because our final exam has to be a common final.” Consequently, she reported the need to modify the tailored MMH content to comply with the exam.

Collaborating with implementation support practitioners and the health coordinators aided in problem-solving delivery challenges, teachers feeling supported and confident in their approach, and enhanced efficiency with classroom time. “(Before engaging in the implementation supports) I could spend two to three hours just on one lesson (to meet the needs of students), but with the (updated) lessons…sometimes 20 to 30 min is just going through it and make sure I’m abreast of the information.” Another teacher who experienced challenges with making the IF meetings expressed that she felt she would have benefitted from greater interaction, but the personal costs of engaging in the implementation support given her context were difficult to overcome. “(The health coordinator) was awesome…I think if anything, I didn’t have the time to give to her, she was always willing to meet, to help…” This teacher reported that there was an incentive structure to cover other classes during planning time and earning days off rather than engaging with implementation support. “(School leadership) definitely didn’t give me time to work on this curriculum, so I couldn’t take a half day to work on it or anything like that.”

## Discussion

This study examines the cost of deploying implementation strategies to enhance the delivery of a universal intervention (MMH) for drug use prevention. We determine costs across multiple phases of implementation and shareholder perspectives. We also use mixed methods, using qualitative data to explain and expand on the quantitative results. Robust evidence indicates that effective implementation of universal prevention is a good return on investment from a societal perspective [62]. However, effective delivery via deploying implementation strategies requires time and resources that vary across shareholders, and this resource allocation is generally not accounted for when considering costs related to the intervention. An aggregated (e.g., societal) perspective may not provide sufficient information for understanding why implementation fails to achieve desired objectives [16]. Overall, we found that intervention implementation in schools may encounter the “wrong pockets” syndrome whereby some of the program implementation benefits accrue to a system (e.g., state education agency) that did not shoulder the majority of the costs [63, 64]. More proximal economic considerations versus societal-level factors are critical influences on whether or not *individuals* and *organizations* successfully implement and sustain EBIs such as MMH [14]. Thus, we examined the actual costs of implementation, that is, fixed and variable costs, and the level of alignment in relative priorities and resource burdens for implementing drug use prevention across shareholder groups.

The base case per school for deploying Enhanced REP was $11,903, with the bulk of the costs being personnel time costs, consistent with other implementation research [41, 65, 66]. In the current study, this equates to $113/student, assuming a state average of 26.4 students per classroom based on the most recent National Center for Education Statistics available [67]. Estimates of implementation costs vary widely, and benchmarking these costs against implementing similar interventions is challenging. Comprehensive prevention interventions’ costs and implementation are infrequently reported [68]. One study reported, for example, that costs for an intervention similar to MMH was, on average, $188/student (adjusted to 2022 USD) [69]. However, the extent to which implementation costs were fully captured in this estimate is unclear. The field of implementation science will benefit from continuing to build the evidence base for implementation strategy costs to identify better what is considered a low- versus high-cost implementation strategy in a specific setting (e.g., schools) and with a particular population (e.g., all students for universal prevention).

Considering costs by phase, costs were highest during the implementation phase, followed by preparation, exploration, and sustainment. The trial-based analysis indicates that the relative cost burden was highest for the state agency during the preparation phase, as with the coordinating body (i.e., university), RESA cost burden was evenly distributed during the preparation and implementation phases and, as expected, the teacher cost burden was heavily concentrated in the implementation phase. This information can help inform implementation planning and understand resource burdens over time across groups. For example, when engaging in an implementation support effort such as deploying Enhanced REP, the RESA can plan for resource allocation that will vary over time, with it being highest during preparation and implementation. Similarly, teachers can prepare for the expected time costs to ramp up during the implementation phase. These reported costs, however, may not reflect the total costs of implementation, including other costs that may not be fully accounted for in these analyses.

The qualitative results aid in expanding our understanding of some of the costs not fully represented in the analyses. We found that the teacher time cost burden, in some cases, for example, is likely an underestimate. Consistent with other school-based research, teacher time costs, including those outside the education sector (e.g., typical work activities) that may be personal opportunity costs related to time participating in implementation activities, are common [70]. Given the current staffing shortages [71], potential costs associated with the de facto expectation of personal opportunity costs to engage with updated practices can have notable downstream impacts. First, from an economic perspective, this is likely to accelerate staff turnover, particularly as this profession has suffered from attrition for many years, which has only accelerated during COVID-19 [72]. Recruiting and training new staff can be very expensive for organizations. Second, the added stress of high personal costs hampers student motivation and learning, which are essential to implementation success and education’s primary objectives [73, 74].

Our analyses also focus on generating information for decision-makers to inform resource allocation for implementation via scenario analyses and BIA. We estimated cost ranges in the scenario analysis, depending on personnel salary at the lower (10th percentile: $8264/school) and higher (90th percentile: $15,201/school). While our base case analysis uses median wages for each personnel category, assuming a typical labor market, school districts may be subject to differing labor market conditions based on outside factors such as location and experience levels. The results from these analyses can support comparing multiple scenarios that reflect potential uncertainties in cost estimates in a reliable manner, given the pilot study parameters. The scenario analysis information can aid educational systems in estimating possible costs and ranges based on their staff experience levels and salary ranges. We also examined the budget impact of Enhanced REP replication in other school settings. While there are varied applications of budget impact analysis, we apply BIA pragmatically consistent with implementation research. The BIA provides information on how adopting Enhanced REP may influence an organization, system, or individual from a budgetary perspective [51]. It aids in answering a central question related to implementation: “What will it cost us?” We found that most variable costs would be offset by the RESA, the agency that services school districts in their region and provides implementation support, including specifically for MMH. Replication cost estimates indicate that the additional cost per school to deploy Enhanced REP would be $8659. The BIA provides an initial assessment of replication for Enhanced REP. Significantly, this information will aid implementation shareholders and decision-makers across levels, including state agencies and implementers (Fig. 1), in allocating sufficient resources to set a foundation for implementation success. Resources to support implementation strategy deployment can be viewed as “enabling” in that they are *foundational* to successful intervention implementation [75]. Thus, estimating the resources needed and associated costs is a vital first step to effective implementation. Qualitative results expand our understanding of various costs not sufficiently represented in the analysis (e.g., personal opportunity costs) and may be important considerations when estimating future potential budget impacts.

Schools are highly heterogeneous settings. Qualitative results indicate that who takes responsibility for various tasks may vary by district or region. For example, when implementers are asked to adopt a new or updated intervention or have a need to engage with implementation supports (e.g., IF from health coordinators), the extent to which this results in personal time costs, that is, opportunity costs, and highly dependent on the building leadership. If the task is to adjust to new population needs and engage in activities to meet that need, we found that school buildings and school leadership can either (a) adjust implementers’ responsibilities and provide flexibility to meet these needs, which may be at a “cost” to the building-level priorities such as covering classes with absent teachers, or (b) require or otherwise strongly incentivize the teachers to assist with using flexible time to meet building-level needs, but with a higher personal opportunity cost. The latter relies heavily on the implementers’ inclination to meet the needs of students at a notable personal cost. Thus, our reported costs at the implementer level likely reflect an underestimate without considering sufficiently the additional costs related to implementation strategy deployment, specifically time costs outside the education sector.

Collectively, our results indicate that collaboration and support within school buildings and across levels notably impact the capacity for effective implementation from the teachers’ perspective. Shared norms and expectations of the importance of MMH implementation, also called strategic implementation climate [61], emerged as an essential factor influencing implementation alignment and efficiency. A building-level climate that was not supportive of EBI implementation (i.e., MMH) and inconsistent with school priorities or expectations resulted in reduced reports of MMH delivery and challenges to engaging with IF, despite a supportive system (e.g., RESA, implementation support professionals). According to our qualitative results, this resulted in notable inefficiencies in Enhanced REP deployment. Researchers have established a link between molar school climate and student outcomes [76, 77]. Our qualitative results indicate that more research is needed to examine the strategic implementation climate related to support for implementing a specific intervention within a school building to deploy implementation strategies effectively and efficiently [61].

Our mixed methods results also indicate the need to enhance focus on building-level implementation costs and priorities. For example, in the school where the priorities and resources were more aligned, there were also likely other consequential costs that were not included, such as additional staffing costs to protect the health teacher’s planning and PD time. While we did account for some additional staffing costs (e.g., substitutes during training), there were likely additional costs and priorities at the building level. While the default interested parties were included in the current analysis, our results suggest a significant gap in some contexts in costs and priorities. Future research would benefit from a more explicit focus on exploring the building-level costs and benefits in the context of implementer-level costs.

### Limitations

While we estimated costs related to the implementation strategy within the education sector and outside (e.g., personal time costs) when these could be reasonably estimated for this pragmatic evaluation, this did not fully capture all costs. Teacher time costs do not fully reflect the opportunity cost of time to participate in Enhanced REP as we could not capture the full scope of all such costs. While important, these costs are also complex and challenging to capture due to the high participant burden. The costs relate to time costs potentially diverted from other work activities that can impact productivity and outside work (e.g., impact on personal time costs). However, pragmatic costing of implementation studies requires a balance between accurate estimates and shareholder burden. In the current pilot study, it was not feasible to estimate all costs comprehensively at all levels. The level of accuracy of reported costs, especially the non-IF costs, may vary as some of the costs were based on recall of past events. We focused on greater precision with the IF costs within the education sector, using weekly time logs, as this was a demonstrated influential parameter in previous research [65].

As this was a pilot study, our number of participants may not be sufficient to estimate the Enhanced REP deployment with scale-up accurately. The scenario analysis and BIA address this limitation by providing information about costs in different scenarios and with replication. This analysis intentionally has a limited scope to benefit decision-makers and other partners most. Still, it does not reflect the aggregate societal perspective regarding costs and resources. While we estimated sustainment costs based on the pilot study’s experimental design, the sustainment costs during a full-scale trial may vary from this estimate. Generally, sustainment costs are very underdeveloped. Thus, this study aims to add to the cost of sustainment evidence base. These results may not be generalizable to other settings but can inform assessing implementation strategy cost estimates in schools. Finally, we experienced attrition at the school level, partly because we conducted the study during the COVID-19 pandemic, which may influence our estimates of Enhanced REP costs. We were, however, due to including multiple teachers over multiple semesters, and through incorporating qualitative data, able to make reasonable estimates of Enhanced REP costs despite the attrition. Also, our *t*-tests of school-level characteristics do not indicate heterogeneity between the trial and control group analytic sample based on racial demographics or socioeconomic status. Despite these limitations, our cost estimates provide a starting point for estimating Enhanced REP costs as part of a more extensive study. Finally, an essential next step in this research is calculating the potential return on investment for implementation strategy deployment using methods of comparative economic evaluation such as cost-effectiveness or cost–benefit analysis. However, estimating costs each shareholder group bears is a vital first step in successfully accounting for resources needed with implementation strategy deployment.

## Conclusions

Educational settings are central for providing health-focused interventions for children and youth, yet many implementation and sustainment efforts fail due to limited attention to the costs and resources required. Information on implementation strategy costs and local budget impacts from multiple perspectives is essential to data-driven decision-making about resource allocation for successful EBI delivery, including within educational settings. This mixed methods study identified the costs and priorities of deploying Enhanced Replicating Effective Programs (REP) to implement the Michigan Model for Health™, a universal school-based prevention intervention, from key shareholder perspectives. Additional work is needed to enhance alignment in costs and resources across perspectives for successful implementation and achieving intervention public health impact.

### Supplementary Information

**Additional file 1. **Testing for Heterogeneity of School Randomization.

## Data Availability

The qualitative datasets generated and analyzed during the current study are not publicly available, given the need for specific context to grasp their usefulness. However, code reports from the qualitative interviews and deidentified data are available from the corresponding author on reasonable request.

## Abbreviations

BIA: Budget impact analysis
BLS: Bureau of Labor Statistics
CASEL: The Collaborative on Academic and Social and Emotional Learning
COINS: Cost of Implementing New Strategies
EBI: Evidence-based intervention
EPIS: Exploration, Preparation, Implementation, Sustainment framework
IF: Implementation facilitation
iPARIHS: Integrated Promoting Action on Research Implementation in Health Services framework
MDE: Michigan Department of Education
MDHHS: Michigan Department of Health and Human Services
MMH: Michigan Model for Health™
PD: Professional development
RCT: Randomized controlled trial
REP: Replicating Effective Programs
RESA: Regional Education Service Agency

## References

[1] Herlitz L, MacIntyre H, Osborn T, Bonell C (2020). The sustainability of public health interventions in schools: a systematic review. Implementat Sci.

[2] Shoesmith A, Hall A, Wolfenden L, Shelton RC, Powell BJ, Brown H (2021). Barriers and facilitators influencing the sustainment of health behaviour interventions in schools and childcare services: a systematic review. Implementation Sci.

[3] Gold HT, McDermott C, Hoomans T, Wagner TH (2022). Cost data in implementation science: categories and approaches to costing. Implement Sci.

[4] O’Leary M, Hassmiller Lich K, Frerichs L, Leeman J, Reuland D, Wheeler S (2022). Extending analytic methods for economic evaluation in implementation science. Implementation Sci.

[5] Saldana L, Ritzwoller D, Campbell M, Block E (2022). Using economic evaluations in implementation science to increase transparency in costs and outcomes for organizational decision-makers. Implement Sci Commun.

[6] Wagner T, Yoon J, Jacobs J, So A, Kilbourne M, Yu W (2020). Estimating Costs of an Implementation Intervention. Med Decis Making.

[7] Powell B, Fernandez M, Williams N, Aarons G, Beidas RS, Lewis C (2019). Enhancing the impact of implementation strategies in healthcare: a research agenda. Front Public Health.

[8] Proctor EK, Silmere H, Raghavan R, Hovmand P, Aarons G, Bunger A (2011). Outcomes for implementation research: conceptual distinctions, measurement challenges, and research agenda. Adm Policy Ment Health.

[9] Raghavan R, Brownson RC, Colditz GA, Proctor EK (2018). The role of economic evaluation in dissemination and implementation research. Dissemination and implementation research in health: translating science to practice.

[10] Bauer M, Damschroder L, Hagedorn H, Smith J, Kilbourne A (2015). An introduction to implementation science for the non-specialist. TT - BMC Psychology.

[11] Kilbourne A, Neumann MS, Pincus HA, Bauer MS, Stall R (2007). Implementing evidence-based interventions in health care: application of the replicating effective programs framework. Implement Sci.

[12] Kilbourne A, Almirall D, Eisenburg D, Waxmonsky J, Goodrich DE, Fortney JC (2014). Adaptive Implementation of Effective Programs Trial (ADEPT): cluster randomized SMART trial comparing a standard versus enhanced implementation strategy to improve outcomes of a mood disorders program. Implement Sci.

[13] Kilbourne A, Smith SN, Choi SY, Koschmann E, Liebrecht C, Rusch A (2018). Adaptive School-based Implementation of CBT (ASIC): clustered-SMART for building an optimized adaptive implementation intervention to improve uptake of mental health interventions in schools. Implement Sci.

[14] Eisman A, Kilbourne A, Dopp A, Saldana L, Eisenberg D (2020). Economic evaluation in implementation science: making the business case for implementation strategies. Psychiatry Res.

[15] Ritchie MJ, Dollar KM, Kearney LK, Kirchner JE (2014). Research and services partnerships: responding to needs of clinical operations partners: transferring implementation facilitation knowledge and skills. PS.

[16] Eisman AB, Quanbeck A, Bounthavong M, Panattoni L, Glasgow RE (2021). Implementation science issues in understanding, collecting, and using cost estimates: a multi-stakeholder perspective. Implement Sci.

[17] Drummond M, Sculpher M, Claxton, K, Stoddart G, Torrance G. Methods for the economic evaluation of health care programmes. Oxford ; New York: Oxford University Press; 2015. 379 p. (Oxford medical publications).

[18] Ferlie EB, Shortell SM (2001). Improving the quality of hhealth care in the United Kingdom and the United States: a framework for change. Milbank Q.

[19] Barrett C, Pas E (2020). A cost analysis of traditional professional development and coaching structures in schools. Prev Sci.

[20] Jackson K, Fixsen D, Ward C, Waldroup A, Sullivan V. Accomplishing effective and durable change to support improved student outcomes. Chapel Hill, NC: University of North Carolina; 2018 Jun. (State Implementation and Scaling-up of Evidence-based Practices (SISEP)).

[21] Bronfenbrenner U, Gauvain M, Cole M (2005). Ecological models of human development. Readings on the Development of Children.

[22] Dopp A, Mundey P, Beasley L, Silovsky J, Eisenberg D. Mixed-method approaches to strengthen economic evaluations in implementation research. Implementation Science. 2019;14(2). Available from: https://implementationscience.biomedcentral.com/articles/10.1186/s13012-018-0850-6. [cited 2019 Mar 5]. 10.1186/s13012-018-0850-6 PMC6329154 30635001

[23] Marmot M (2005). Social determinants of health inequalities. Lancet.

[24] LeTendre M, Reed M (2017). The effect of adverse childhood experience on clinical diagnosis of a substance use disorder: results of a nationally representative study. Subst Use Misuse.

[25] Neumann M, Sogolow E (2000). Replicating effective programs: HIV/AIDS prevention technology transfer. AIDS Educ Prev.

[26] Bandura A. Social learning theory. Prentice-Hall; 1977. Available from: http://mirlyn.lib.umich.edu/Record/000313256 CN - LB1084 .B3571.

[27] Rogers E. Diffusion of innovations. 2003. Available from: http://mirlyn.lib.umich.edu/Record/004335364 CN - HM 101 .R72 2003

[28] Harvey G, Kitson A (2015). PARIHS revisited: from heuristic to integrated framework for the successful implementation of knowledge into practice. Implement Sci.

[29] Kilbourne A, Abraham KM, Goodrich DE, Bowersox NW, Almirall D, Lai Z (2013). Cluster randomized adaptive implementation trial comparing a standard versus enhanced implementation intervention to improve uptake of an effective re-engagement program for patients with serious mental illness. Implement Sci.

[30] Lee R, Gortmaker S, Brownson R, Colditz G, Proctor E (2018). Health Dissemination and Implementation within Schools. Dissemination and implementation research in health: translating science to practice.

[31] Shope J, Copeland L, Maharg R, Dielman T (1996). Effectiveness of a high school alcohol misuse prevention program. Alcohol Clin Exp Res.

[32] O’Neill J, Clark J, Jones J (2011). Promoting mental health and preventing substance abuse and violence in elementary students: a randomized control study of the michigan model for health. J Sch Health.

[33] Bandura A (1989). Human agency in social cognitive theory. Am Psychol.

[34] Rosenstock IM (1974). Historical Origins of the Health Belief Model. Health Educ Monogr.

[35] CASEL. Program Guide. 2022. Program Guide CASEL: Collaborative for Academic Social and Emotional Learning. Available from: https://pg.casel.org/. [cited 2022 Feb 8].

[36] CDC. CDC Healthy Schools. 2019. National Health Education Standards. Available from: https://www.cdc.gov/healthyschools/sher/standards/index.htm. [cited 2021 Dec 21].

[37] Rockhill S. Use of the Michigan Model for Health Curriculum among Michigan Public Schools: 2017. In: Lansing MI, editor. Michigan Department of Health and Human Services, Lifecourse Epidemiology and Genomics Division, Child Health Epidemiology Section. 2017.

[38] Eisman AB, Kilbourne AM, Ngo Q, Fridline K, Zimmerman MA, Greene D (2020). Implementing a state-adopted high school health curriculum: a case study. J Sch Health.

[39] Durlak J, Weissberg R, Pachan M (2010). A meta-analysis of after-school programs that seek to promote personal and social skills in children and adolescents. Am J Community Psychol.

[40] Aarons G, Hurlburt M, Horwitz S (2011). Advancing a conceptual model of evidence-based practice implementation in public service sectors. Adm Policy Ment Health.

[41] Saldana L, Chamberlain P, Bradford W, Campbell M, Landsverk J (2014). The cost of implementing new strategies (COINS): a method for mapping implementation resources using the stages of implementation completion. Child Youth Serv Rev.

[42] Neumann PJ, Sanders GD, Russell LB, Siegel JE, Ganiats TG. Cost-Effectiveness in Health and Medicine. Oxford University Press; 2016. 537 p.

[43] Michigan - May 2021 OEWS State Occupational Employment and Wage Estimates. Available from: https://www.bls.gov/oes/current/oes_mi.htm. [cited 2023 Feb 16].

[44] Mackinac Center. Michigan Government Salaries Database. Available from: https://www.mackinac.org/salaries?report=education&search=&sort=wage2022-desc. [cited 2023 Feb 16].

[45] U.S. Department of Labor. U.S. Bureau of Labor Statistics. 2023. BLS Wage Data by Area and Occupation. Available from: https://www.bls.gov/bls/blswage.htm. [cited 2023 Jul 12].

[46] State of Michigan. ORS- Public School Reporting Units. K-12 and ISD teachers and other employees paid by contract. Available from: https://www.michigan.gov/psru/reporting-resources/reporting-instruction-manual/5-reporting-hours-for-service-credit/5-05-k-12-and-isd-teachers-and-other-employees-paid-by-contract. [cited 2023 Sep 6].

[47] Bowser D, Henry B, McCollister K (2021). Cost analysis in implementation studies of evidence-based practices for mental health and substance use disorders: a systematic review. Implement Sci.

[48] Simoens S (2009). Health Economic Assessment: A Methodological Primer. Int J Environ Res Public Health.

[49] Drummond M, Coyle D (1998). The role of pilot studies in the economic evaluation of health technologies. Int J Technol Assess Health Care.

[50] Ryan DA, Montoya ID, Koutoujian PJ, Siddiqi K, Hayes E, Jeng PJ (2023). Budget impact tool for the incorporation of medications for opioid use disorder into jail/prison facilities. J Subst Use Addict Treat.

[51] Wagner TH, Dopp AR, Gold HT (2020). Estimating Downstream Budget Impacts in Implementation Research. Med Decis Making.

[52] Hamilton, AB. Qualitative methods in rapid turn-around health services research. Veterans Affairs Health Services Research & Development Cyberseminar presented at; 2013.

[53] Sobo EJ, Simmes DR, Landsverk JA, Kurtin PS (2003). Rapid assessment with qualitative telephone interviews: lessons from an evaluation of California’s Healthy Families Program & Medi-Cal for Children. Am J Eval.

[54] Taylor B, Henshall C, Kenyon S, Litchfield I, Greenfield S (2018). Can rapid approaches to qualitative analysis deliver timely, valid findings to clinical leaders? A mixed methods study comparing rapid and thematic analysis. BMJ Open.

[55] Koenig C, Abraham T, Zamora K, Hill C, Kelly PA, Uddo M (2016). Pre-implementation strategies to adapt and implement a veteran peer coaching intervention to improve mental health treatment engagement among rural veterans: Pre-implementation of a Mental Health Intervention. J Rural Health.

[56] Terzis LD, Saltzman LY, Logan DA, Blakey JM, Hansel TC (2022). Utilizing a matrix approach to analyze qualitative longitudinal research: a case example during the COVID-19 pandemic. Int J Qual Methods.

[57] Miles MB, Huberman AM, Saldaña J. Qualitative data analysis: a methods sourcebook. Third edition. Thousand Oaks, California: SAGE Publications, Inc; 2014. 381 p.

[58] Creswell J, Plano Clark V. Designing and conducting mixed methods research. Third Edition. Los Angeles : SAGE, [2018]; 2018. 492 p.

[59] Center for Educational Performance and Information (CEPI). MI School Data: Michigan’s Official Education Data Source. 2022 [cited 2023 Oct 27]. Student Enrollment Counts Report. Available from: https://www.mischooldata.org/student-enrollment-counts-report/.

[60] Ehrhart M, Aarons G, Farahnak L (2014). Assessing the organizational context for EBP implementation: the development and validity testing of the Implementation Climate Scale (ICS). Implement Sci.

[61] Lyon A, Cook C, Brown E, Locke J, Davis C, Ehrhart M (2018). Assessing organizational implementation context in the education sector: confirmatory factor analysis of measures of implementation leadership, climate, and citizenship. Implementation Sci.

[62] U.S. Department of Education. Prevalence and implementation fidelity of research-based prevention programs in public schools: final report. Washington, D.C.: U.S. Department of Education, Office of Planning, Evaluation and Policy Development, Policy and Programs Study Service; 2011. Report No.: ED-00-CO-0119.

[63] Butler S. How “Wrong Pockets” Hurt Health. JAMA Forum Archive. 2018 Aug 22;A7(1). Available from: 10.1001/jamahealthforum.2018.0033. [cited 2023 Mar 16].

[64] Humphreys K, Wagner TH, Gage M (2011). If substance use disorder treatment more than offsets its costs, why don’t more medical centers want to provide it? A budget impact analysis in the Veterans Health Administration. J Subst Abuse Treat.

[65] Eisman AB, Hutton DW, Prosser LA, Smith SN, Kilbourne AM (2020). Cost-effectiveness of the Adaptive Implementation of Effective Programs Trial (ADEPT): approaches to adopting implementation strategies. Implement Sci.

[66] Salloum R, D’Angelo H, Theis R, Rolland B, Hohl S, Pauk D (2021). Mixed-methods economic evaluation of the implementation of tobacco treatment programs in National Cancer Institute-designated cancer centers. Implement Sci Commun.

[67] National Center for Education Statistics. National Teacher and Principal Survey (NTPS). National Center for Education Statistics; 2018. Available from: https://nces.ed.gov/surveys/ntps/tables/ntps1718_fltable06_t1s.asp.[cited 2023 Oct 4].

[68] Ekwaru JP, Ohinmaa A, Dabravolskaj J, Maximova K, Veugelers PJ (2021). Cost-effectiveness and return on investment of school-based health promotion programmes for chronic disease prevention. Eur J Pub Health.

[69] Aos S, Lee S, Drake E, Pennucci A, Klima T, Miller M, et al. Return on investment: Evidence-based options to improve statewide outcomes. Olympia, WA: Washington State Institute for Policy Research. Olympia, WA: Washington State Institute for Policy Research; 2011.

[70] But at What Cost? Teacher Mental Health during COVID-19. Pandemic Research Report. Canadian Teachers’ Federation. Canadian Teachers’ Federation; 2022. Available from: https://proxy.lib.wayne.edu/login?url=https://www.proquest.com/reports/at-what-cost-teacher-mental-health-during-covid/docview/2722475286/se-2?accountid=14925

[71] García E, Weiss E. Economic Policy Institute. The teacher shortage is real, large and growing, and worse than we thought: The first report in “The Perfect Storm in the Teacher Labor Market” series. Available from: https://www.epi.org/publication/the-teacher-shortage-is-real-large-and-growing-and-worse-than-we-thought-the-first-report-in-the-perfect-storm-in-the-teacher-labor-market-series/. [cited 2023 Mar 16].

[72] Pressley T (2021). Factors contributing to teacher burnout during COVID-19. Educ Res.

[73] Shen B, McCaughtry N, Martin J, Garn A, Kulik N, Fahlman M (2015). The relationship between teacher burnout and student motivation. Br J Educ Psychol.

[74] Sutcher L, Darling-Hammond L, Carver-Thomas D (2019). Understanding teacher shortages: An analysis of teacher supply and demand in the United States. Education Policy Analysis Archives.

[75] Nilsen P, Bernhardsson S (2019). Context matters in implementation science: a scoping review of determinant frameworks that describe contextual determinants for implementation outcomes. BMC Health Serv Res.

[76] Thapa A, Cohen J, Guffey S, Higgins-D’Alessandro A (2013). A review of school climate research. Rev Educ Res.

[77] Johnson B, Stevens JJ (2006). Student achievement and elementary teachers’ perceptions of school climate. Learning Environ Res.

