# Supplementary material for: A mixed methods partner-focused cost and budget impact analysis to deploy implementation strategies for school-based prevention
Source: Implement Sci Commun. 2023 Nov 9;4:133. doi: 10.1186/s43058-023-00511-6 (PMC10636820; doi:10.1186/s43058-023-00511-6)
Supplement: Supplementary file 1 — Additional file 1. Testing for Heterogeneity of School Randomization. [file 43058_2023_511_MOESM1_ESM.docx]

**Appendix**

**Testing for Heterogeneity of School Randomization**

| **School Demographics** | **Mi-LEAP** | **Control** | **P-Value** |
| --- | --- | --- | --- |
| Number of schools | 4 | 5 | - |
| Average school size | 1018.25 | 1062.60 | 0.922 |
| Average free-and-reduced lunch rate | 42.79% | 41.11% | 0.395 |
| Number of schools with high free-and-reduced lunch rate | 2 | 1 | - |
| White | 51.82% | 63.67% | 0.613 |
| Black | 37.62% | 26.48% | 0.663 |
| LatinX | 4.14% | 3.42% | 0.601 |
| Asian | 3.25% | 1.56% | 0.587 |
| Native American | 0.02% | 0.02% | 0.587 |
| Mixed | 5.76% | 2.91% | 0.132 |
| American Indian or Alaska Native | 0.22% | 0.18% | 0.757 |
| Native Hawaiian or other Pacific Islander | 0.02% | 0.06% | 0.234 |

Source: *MI School Data* [59]. Mi-LEAP refers to the treatment schools in the randomization. The P-Value reported corresponds to a Student T-Test for statistical differences between the treatment and control samples.
